# Enhanced vitamin E content in an Indica rice cultivar harbouring two transgenes from *Arabidopsis thaliana* involved in tocopherol biosynthesis pathway

**DOI:** 10.1111/pbi.13578

**Published:** 2021-03-21

**Authors:** Sathish Sundararajan, Venkatesh Rajendran, Hari Priya Sivakumar, Safia Nayeem, Harish Mani Chandra, Ashutosh Sharma, Sathishkumar Ramalingam

**Affiliations:** ^1^ Plant Genetic Engineering Laboratory Department of Biotechnology Bharathiar University Coimbatore India; ^2^ Department of Biotechnology Thiruvalluvar University Serkkadu Vellore India; ^3^ Technologico de Monterrey Centre of Bioengineering Santiago de Querétaro Queretaro Mexico

**Keywords:** *Agrobacterium*, rice, vitamin E, HPLC

Micronutrient deficiency results in malnutrition, which is prevalent all over the world and may lead to premature death in women and children (White and Broadley, 2009). Strategies formulated earlier including supplementation and fortified foods were not successful, owing to socio‐economic and technical hurdles (Mayer *et al.,* 2008). The subsequently evolved strategy of biofortification is a viable biotechnological tool to achieve desired results without compromising the agronomical values of crops. Conferring the genetic trait to improve vital nutrient accumulation in the edible parts of staple food crops, such as rice, through metabolic engineering is considered a fast, sustainable and cost‐effective alternative to conventional breeding (Maestre *et al.,* 2017). In earlier reports from our laboratory, enhanced α‐tocopherol levels in the stable transformants of *Nicotiana tabacum* (Harish *et al.,* 2013a, 2013b) and in *Nicotiana benthamiana* adopting a transient expression system using *A. thaliana* tocopherol cyclase *(TC) and* homogentisate phytyl transferase *(HPT)* (Sathish *et al.,* 2018) were shown. In the present study, *Agrobacterium*‐mediated transformation of *Indica* rice ASD16 with two genes involved in tocopherol biosynthesis, *viz*., *TC* and *HPT,* was carried out and the transgenic plants were analysed for the vitamin E (α‐tocopherol) content.

Mature seed‐derived embryogenic calli were subjected to genetic transformation (Sundararajan *et al.,* 2020) by employing two gene constructs, *viz*., pCAMBIA 1305.1 harbouring *TC* and pNutKan harbouring *HPT* (Fig 1a), individually, and in a co‐transformation system, the calli were infected with both gene constructs. Putative transgenic plants recovered from all three independent experiments were established in a containment facility and no detectable morphological variations were found between the acclimatized NT and the transgenic plants (Fig 1b). Ten plants from all three experiments were taken for initial PCR analysis with the marker genes respective to each gene construct, that is *hpt*II in pCAMBIA 1305.1 *TC* and *npt*II in pNutKan *HPT*. Nine plants harbouring *TC*, 6 plants harbouring *HPT* and 7 *TC + HPT* plants showed positive PCR amplification of the marker genes (data not shown).

**Figure 1:**
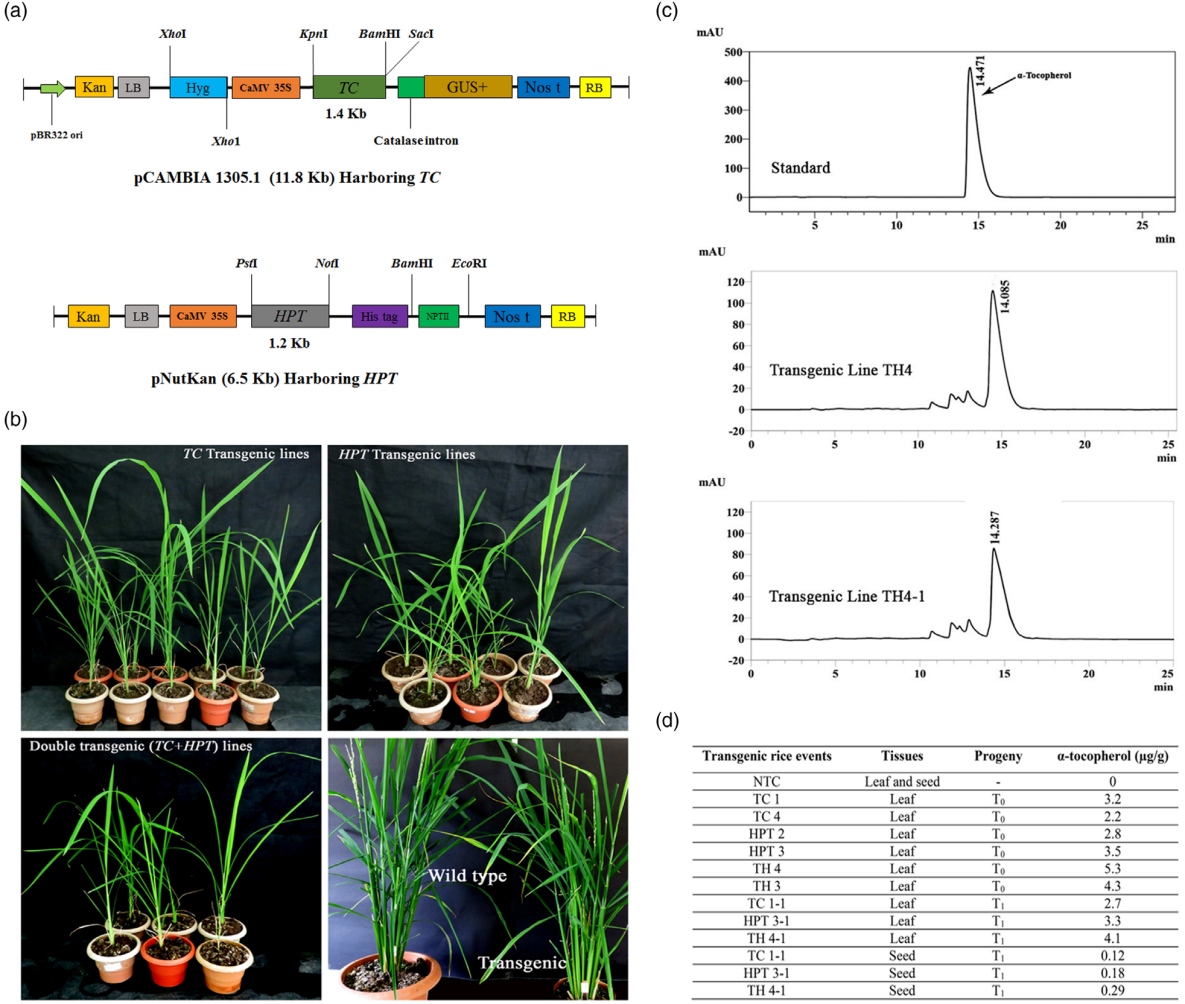
1(a) Maps of binary vectors harbouring *TC* and *HPT* mobilized into *Agrobacterium* strain LBA4404; (b) transgenic lines recovered from genetic transformation experiments hardened in plant containment facility; (c) representative chromatograms of HPLC analysis in T_0_ and T_1_ lines harbouring *TC* and *HPT*; and (d) quantification of α‐tocopherol in T_0_ and T_1_ transgenic rice.

A preliminary GUS histochemical assay for the transgenic lines generated with *TC* and *TC + HPT* revealed the formation of blue colour in the leaves and roots of transgenic plants confirming the presence of the *GUS* gene, whereas no blue coloration was detected in the NT control. PCR analysis of the transgenic plants harbouring individual *TC* and *HPT* showed the expected band sizes (1.2 Kb for *HPT* and 1.4 Kb for *TC*). With the *TC + HPT* plants, amplification of both selectable marker genes during the initial PCR analysis was observed. However, in the PCR using gene‐specific primers, among the seven plants, only five showed the presence of both the transgenes (plants subsequently renamed as TH1 to TH5). Southern blot analysis revealed the expected hybridization signals corresponding to *HPT* (1.2 kb) in all the six plants digested with *Pst*I and *Not*I, and *TC* (1.4 kb) in all the nine plants digested with *Kpn*I and *Bam*HI. For the *TC + HPT* lines, two individual blots were prepared using genomic DNA digested with *Kpn*I and *Bam*HI for *TC* and *Pst*I and *Not*I for *HPT*. The *HPT*‐probed blot showed a corresponding hybridization signal of 1.2 kb, and a hybridization signal corresponding to 1.4 kb of the *TC* gene was observed in the *TC*‐probed blot in all the five transgenic lines demonstrating the stable integration of the transgenes in the plants (data not shown).

Transcript analysis indicated that among the *TC* plants, TC3, TC4 and TC7 showed significantly higher levels of transgene expression (19.7‐fold, 21‐fold and 20.2‐fold, respectively) as compared to the control. The relative gene expression of *HPT* lines was found to be higher as compared to *TC* lines. The quantum increase ranged from 9.5 to 17.0 among *HPT* lines with HPT4 showing the highest gene expression (26.3‐fold) followed by HPT5 (23.6‐fold increase). Among the *TC + HPT* lines, TH4 showed maximum gene expression to the tune of 26.7‐fold (*HPT*) and 32.5‐fold (*TC*) (data not shown).

Two plants each harbouring *TC*, *HPT* and *TC + HPT* were analysed for α‐tocopherol content in the transgenic leaves by HPLC. The determination of α‐tocopherol in the samples was based on peaks observed at 295 nm with a UV detection lamp. The retention time of the metabolite was confirmed by injecting the authentic standard (Sigma‐Aldrich, St. Louis, MO), and the quantification in transgenic lines was performed accordingly. Results showed that among the *TC* lines, TC1 showed a 3.26‐fold and TC4 showed a 2.2‐fold increase in α‐tocopherol. The *HPT* lines showed relatively higher α‐tocopherol content as compared to that of the *TC* lines, wherein HPT3 showed a 3.5‐fold and HPT2 showed a 2.8‐fold increase in α‐tocopherol. The *TC + HPT* plants exhibited the highest α‐tocopherol content among all the transgenic lines. Accordingly, the line TH4 showed a 5.3‐fold increase in α‐tocopherol followed by TH3 (4.3‐fold). Subsequently, three lines (TC1, HPT3 and TH4) were chosen for T_1_ analysis where transgenic plants germinated from T_0_ seeds showed a segregation ratio of 3:1 confirming the Mendelian pattern of inheritance. Ten samples for each progeny were checked by PCR, and the progenies showed respective positive amplicons for the *TC* (1.4 kb) and *HPT* (1.2 kb) and presence of both the genes in the *TC + HPT* progenies (data not shown).

Subsequently, Southern blot analysis revealed the stable integration of the transgenes in the T_1_ progenies. Accordingly, the hybridization signals corresponding to the expected sizes of transgenes were detected in all the samples and no hybridization signal was detected in the NT control. qRT‐PCR analysis of the two randomly selected PCR‐positive plants from each gene construct revealed comparable results as that of the T_0_ plants. The *TC + HPT* lines showed higher relative gene expression as compared to that of the lines that harbour only one gene. Both TC1 progenies showed a comparable increase to the tune of 8.36‐fold (TC 1‐1) and 11.6‐fold (TC 1‐2), and HPT4 progenies showed a significant increase of 11.26‐fold (HPT 4‐1) and 8.95‐fold (HPT 4‐2). The *TC + HPT* progeny TH4 showed the highest relative gene expression about 15.35 (TH 4‐1) and 14.80 (TH 4‐2) folds (data not shown). A sample each from T_1_ progeny that carried *TC, HPT* and *TC + HPT* were analysed for their α‐tocopherol content. Results revealed that TC1‐1 showed a 2.7‐fold increase, HPT3‐1 showed an increase of 3.3‐fold and the *TC + HPT* progeny TH4‐1 showed a 4.1‐fold increase in α‐tocopherol content in the transgenic leaves similar to that of the T_0_ progenies where the *TC + HPT* line showed the highest α‐tocopherol as compared to the lines harbouring either *TC* or *HPT* (Fig. 1c, d). The transgenic T_1_ seeds from the selected progenies were assessed for their α‐tocopherol content and a 0.29‐fold increase in the *TC + HPT* line (TH4‐1) was observed as compared to TC1‐1 (0.12‐fold) and HPT3‐1 (0.18‐fold). Enhanced level of vitamin E in numerous plant species including *A*. *thaliana* leaves (4.4‐fold) and seeds (40%), tobacco leaves (5.5‐fold), lettuce (2‐fold), *Brassica napus* (2.7‐fold) and potato tuber (106%) has been reported utilizing various enzymes of the vitamin E biosynthesis pathway (reviewed by Mène‐Saffrané and Pellaud, 2017). Harish *et al.,* (2013a) proposed that in higher plants, co‐expression of *TC* and *HPT* might increase the overall rate of total pathway flux resulting in a higher content of the end products. In summary, successful enhancement of vitamin E in rice was achieved using overexpression of both *TC* and *HPT*. Though the accumulation is not significant in the seeds, improvement with a multi‐gene strategy with appropriate endosperm‐specific promoters might offer an attractive model for vitamin E biofortification in rice, which would immensely benefit human nutrition and health care.

## Conflict of interest

All authors read, approved the manuscript and declare that there is no conflict of interest.

## Author contributions

SS did the experiments and prepared the manuscript. VR and HPS did the experimental analysis. SN contributed to manuscript preparation and data representations. HMC carried out the initial cloning and assisted in manuscript editing. AS contributed to experimental design, technical interpretation and manuscript editing, and SR conceptualized, supervised the research and critically evaluated the manuscript.
